# Comparative transcriptomic analysis of two *Saccharopolyspora spinosa* strains reveals the relationships between primary metabolism and spinosad production

**DOI:** 10.1038/s41598-021-94251-z

**Published:** 2021-07-20

**Authors:** Yunpeng Zhang, Xiaomeng Liu, Tie Yin, Qi Li, Qiulong Zou, Kexue Huang, Dongsheng Guo, Xiaolin Zhang

**Affiliations:** 1 Beijing Key Laboratory of Nutrition and Health and Food Safety, Nutrition and Health Research Institute, COFCO, Beijing, 102209 People’s Republic of China; 2 Qilu Pharmaceutical (Inner Mongolia) Co., Ltd., Hohhot, 010020 Inner Mongolia People’s Republic of China; 3 School of Food Science and Pharmaceutical Engineering, Nanjing Normal University, Nanjing, 210024 Jiangsu People’s Republic of China

**Keywords:** Antimicrobials, Microbial genetics

## Abstract

*Saccharopolyspora spinosa* is a well-known actinomycete for producing the secondary metabolites, spinosad, which is a potent insecticides possessing both efficiency and safety. In the previous researches, great efforts, including physical mutagenesis, fermentation optimization, genetic manipulation and other methods, have been employed to increase the yield of spinosad to hundreds of folds from the low-yield strain. However, the metabolic network in *S. spinosa* still remained un-revealed. In this study, two *S. spinosa* strains with different spinosad production capability were fermented and sampled at three fermentation periods. Then the total RNA of these samples was isolated and sequenced to construct the transcriptome libraries. Through transcriptomic analysis, large numbers of differentially expressed genes were identified and classified according to their different functions. According to the results, *spnI* and *spnP* were suggested as the bottleneck during spinosad biosynthesis. Primary metabolic pathways such as carbon metabolic pathways exhibited close relationship with spinosad formation, as pyruvate and phosphoenolpyruvic acid were suggested to accumulate in spinosad high-yield strain during fermentation. The addition of soybean oil in the fermentation medium activated the lipid metabolism pathway, enhancing spinosad production. Glutamic acid and aspartic acid were suggested to be the most important amino acids and might participate in spinosad biosynthesis.

## Introduction

Spinosyns is a series of macrolide antibiotics consisted of a tetracyclic lactone decorated with an amino sugar, forosamine, and a natural sugar, 2,3,4-*tri*-*O*-methylated-rhamnose^1,2^. Spinosyns was firstly discovered from the soil sample of Caribbean island in 1982^3^. So far, more than 20 spinosyns analogues have been isolated and identified, among which the most effective constituents are spinosyn A and spinosyn D (spinosad)^4^. Spinosad affects the acetylcholine receptors of insects, causing muscle twitch and a sudden death^5^. Moreover, spinosad exhibits low mammalian toxicity and can be easily degraded^2,6^. Taking the advantages of efficiency and safety, it was awarded “The Presidential Green Chemistry Challenge (U.S.A.)” for three times and was permitted to be used on organic food farming in several countries.

Spinosad is the secondary metabolites synthesized by *Saccharopolyspora spinosa*, which is a gram-positive bacterium with high GC content. During the past decades, the whole genome of *S. spinosa* was sequenced and the genes directly related to spinosyns synthesis were characterized^7^. It is proven that spinosyns biosynthesis is extremely associated with glycolysis, fatty acid degradation, polyketone synthesis. Most genes involved in spinosyns biosynthesis are localized in a large genomic region spanning 74 kilo-base in length in *S. spinosa* genome^8–13^. In this ideal genomic region, five large genes, *spnA*, *B*, *C*, *D* and *E*, encode a type I polyketide synthase, which controls the biosynthesis of the carbon skeleton of spinosyns^8^. In 2017, *spnF* was characterized by Jeon et al. as the key enzyme with the catalytic activity of intramolecular [4 + 2]-cycloaddition^14,15^. In addition with *spnJ*, *L* and *M*, the intramolecular carbon–carbon bond is constructed, resulting in the formation of tetracyclic lactone^8,16^. Then the tetracyclic lactone is decorated by *spnG* and *spnP* with the addition of two deoxysugars, d-forosamine and tri-*O*-methyl-l-rhamnose^9,17^. The d-forosamine is synthesized under the control of *spnO*, *N*, *Q*, *R* and *S*^18–20^. The formation of tri-*O*-methyl-l-rhamnose is related to *spnI*, *K* and *H*^21–23^. Additionally, the four genes (*gtt*, *gdh*, *epi* and *kre*) involved in forosamine and rhamnose synthesis played significant roles in spinosyns formation^24,25^.

Despite the great prospect for the application of spinosad, the yield of spinosad in the wild-type *S. spinosa* strain is too low to meet the industrial production demand. The cost of spinosad is still not cheap enough to be accepted by farmers in the most area of the world. Several approaches have been applied to enhance the production of spinosad. Physical and chemical mutagenesis was the most common method for the breeding of spinosad high-yield strains. Nitrosoguanidine (NTG) exposion and N^+^ implantation were regarded as the most useful approaches for mutagenesis^2^. Also, the spinosyns synthetic gene cluster was duplicated in the spinosyns producing strain or expressed in homologous strain to improve the spinosad production^12,23,25–28^. Furthermore, the primary metabolism pathways, including fatty acid degradation, redox controlling and so on, have been considered to have a relationship with the production of spinosad^13,29–31^.

Additionally, though lots of studies such as proteomics and metabolomics have been done on spinosyns biosynthesis process, the linkage between spinosyns formation and primary metabolism network still remained un-revealed^32–36^. In this study, we try to find out the different metabolism processes in wild-type strain *S. spinosa* ATCC_49460 and spinosad high-yield strain *S. spinosa* S3-3. The comparative transcriptomic analysis was carried out, which would provide the direction of genetic engineering in the next step.

## Results

### Strain cultivation and sampling

The *S. spinosa* fermentation period spanned 7 days, which normally included three phases, lag phase, logarithmic phase and stationary phase^31^. In the lag phase, the *S. spinosa* cell exhibited low growth speed and spinosad was hardly produced. From the end of the lag phase, the cell activity was elevated and the spinosad production was initialized. In the following logarithmic phase, the biomass increased rapidly and spinosad accumulated with a relatively high speed intracellularly. Then, as the fermentation stage comes to the stationary phase, the cell growth stopped, while the spinosad biosynthesis continued. In order to seek for the gene transcription level variation during the fermentation periods, the *S. spinosa* ATCC_49460 (wild-type strain) and *S. spinosa* S3-3 (spinosad high-production strain) samples in two batches at late lag phase (the third day), middle logarithmic phase (the fifth day) and early stationary phase (the seventh day) were obtained. Then the yield of spinosad in the samples were evaluated by HPLC, which indicated that the production of spinosad in *S. spinosa* S3-3 was 6–16 times higher than that in *S. spinosa* ATCC_49460 at these three time points (Figure S1).

### Illumina Hiseq sequencing and genome mapping of *S. spinosa* ATCC_49460 and *S. spinosa* S3-3

Twelve RNA-seq libraries were prepared from *S. spinosa* ATCC_49460 and *S. spinosa* S3-3 samples for gene expression level analysis. The number of raw reads ranged from 23,920,094 to 31,023,334 in the 12 libraries. Then the raw data were qualified using Sickle and SeqPrep programs resulting in tens of millions of clean reads (23,229,014 at least and 30,442,026 at most), which indicated that at least 98.12% of the reads were considered to be clean at Q20 level for each of the twelve libraries. Following by the mapping of the sequenced reads with the whole genome shotgun sequence of *S. spinosa* NRRL18395 (accession number NZ_GL877878–NZ_GL877899), more than 86.92% of the clean reads were annotated, which provided a set of precise data for differentially expressed genes analysis (Table 1).

**Table 1:** Statistical analysis of sequence data and genome mapping.

Samples	*S. spinosa* ATCC_49460						*S. spinosa* S3-3					
	1st_3d	1st_5d	1st_7d	2nd_3d	2nd_5d	2nd_7d	1st_3d	1st_5d	1st_7d	2nd_3d	2nd_5d	2nd_7d
Raw reads	29,117,826	24,034,978	28,357,430	29,965,798	31,023,334	29,902,184	29,464,414	28,389,756	30,151,088	23,920,094	27,319,648	29,770,758
Raw bases (bp)	4,396,791,726	3,629,281,678	4,281,971,930	4,524,835,498	4,684,523,434	4,515,229,784	4,449,126,514	4,286,853,156	4,552,814,288	3,611,934,194	4,125,266,848	4,495,384,458
Clean Reads	28,494,064	23,229,014	27,694,578	29,527,524	30,442,026	29,392,994	28,949,358	27,900,818	29,681,478	23,593,160	26,933,704	29,321,562
Clean bases (bp)	3,938,041,375	3,087,355,459	3,852,140,194	4,155,132,257	4,238,725,463	4,005,483,958	4,043,397,388	3,922,305,578	4,124,329,941	2,946,521,047	3,786,155,248	4,069,799,154
Clean Q20 (%)	98.38	98.12	98.42	98.38	98.34	98.38	98.44	98.32	98.36	99.55	98.41	98.42
Clean Q30 (%)	95.04	94.48	95.08	94.98	94.92	94.98	95.12	94.82	94.94	98.35	95.04	95.08
Mapped reads	27,828,043	21,380,398	25,505,372	27,805,601	29,570,979	27,155,600	25,199,313	24,251,967	28,126,466	23,117,814	24,433,605	26,106,735
Mapped reads ratio (%)	97.66	92.04	92.1	94.17	97.14	92.39	87.05	86.92	94.76	97.99	90.72	89.04

### Gene annotation and enrichment

**Figure 1:**
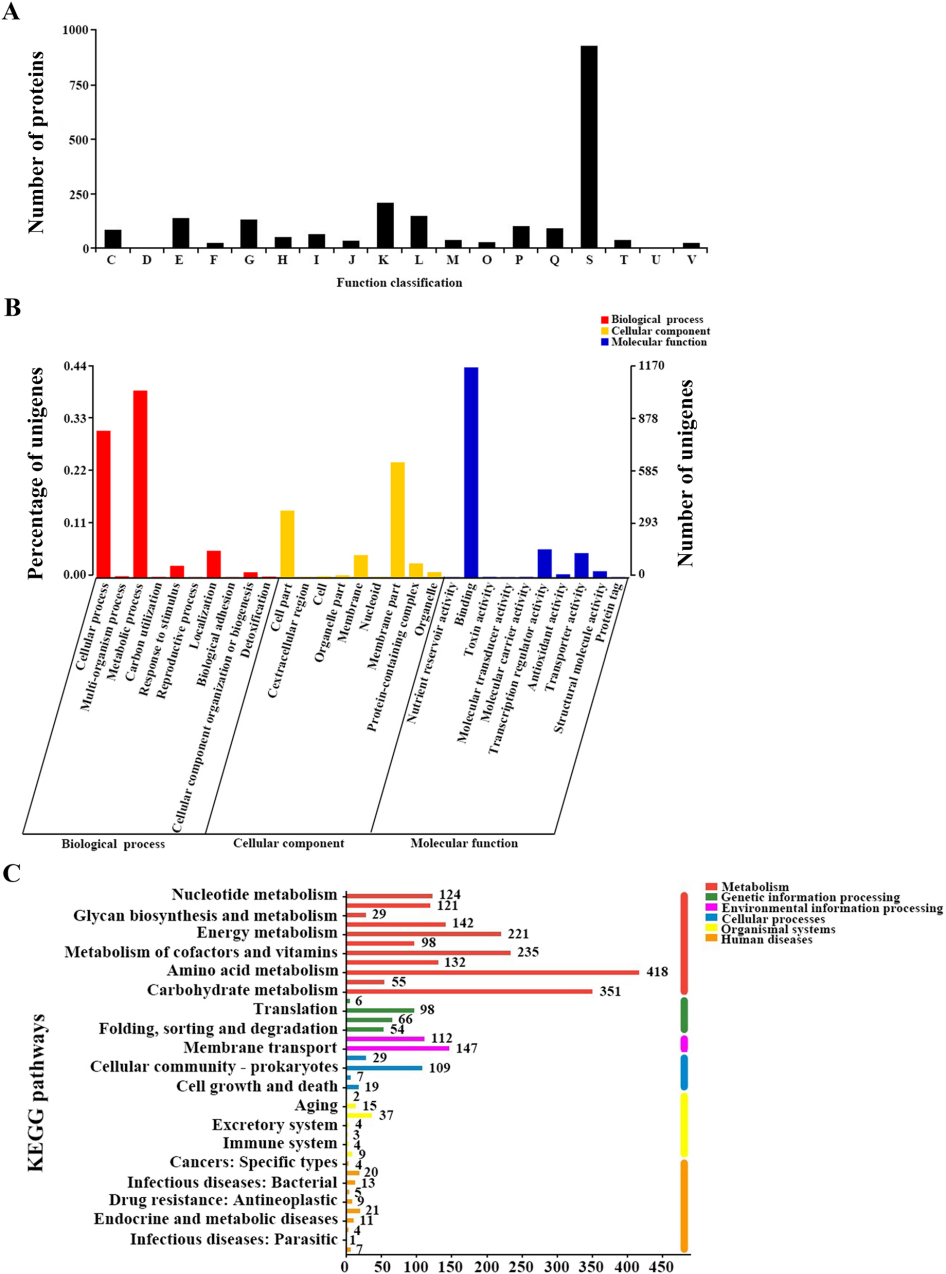
Annotations of the *S. spinosa* genome information using COG database, GO database and KEGG database. (**A**) Annotation of the *S. spinosa* genome sequence using COG database; (**B**) Annotation of the *S. spinosa* genome sequence using GO database; (**C**) Annotation of the *S. spinosa* genome sequence using KEGG database. (The chart was carried out using Majorbio Cloud Platform. URL: http://www.majorbio.com).

To make a clearer understanding of the transcriptomic data, the mapped genes were firstly annotated and classified according to three authoritative databases, Evolutionary genealogy of genes: Non-supervised Orthologous Groups (EggNOG), Gene Ontology (GO) and Kyoto Encyclopedia of Genes and Genomes (KEGG). Through the alignment with the mapped genes in EggNOG database (http://eggnog.embl.de/), 2108 genes were separated into four categories (metabolism, cellular processes and signaling, information storage and processing and poorly characterized), which were subdivided into 18 types. Except for type S (poorly characterized, containing 928 proteins), the most abundant type was type K (transcription, containing 209 proteins), followed by type L (replication, recombination and repair, containing 149 proteins), type E (amino acid transport and metabolism, containing 137 proteins) and type G (carbohydrate transport and metabolism, containing 131 proteins) (Fig. 1A). Based on the GO classification system (http://geneontology.org/), the mapped genes were classified into various sub-categories according to their common characteristics or functions. A total of 2660 mapped genes were classified using the complete set of GO terms into three broad categories: biological process, cellular component and molecular function. And the largest proportion of genes were classified in catalytic activity (1502 genes), followed by binding (1170 genes), metabolic process (1041 genes) and cellular process (817 genes) (Fig. 1B). As for KEGG annotation (http://www.genome.jp/kegg/), metabolism pathway, genetic information processing pathway and environmental information processing pathway were the biggest three pathways, which contained 1238, 222 and 257 genes, respectively (Fig. 1C).

### Characterization of genes specially expressed in *S. spinosa* S3-3

**Figure 2:**
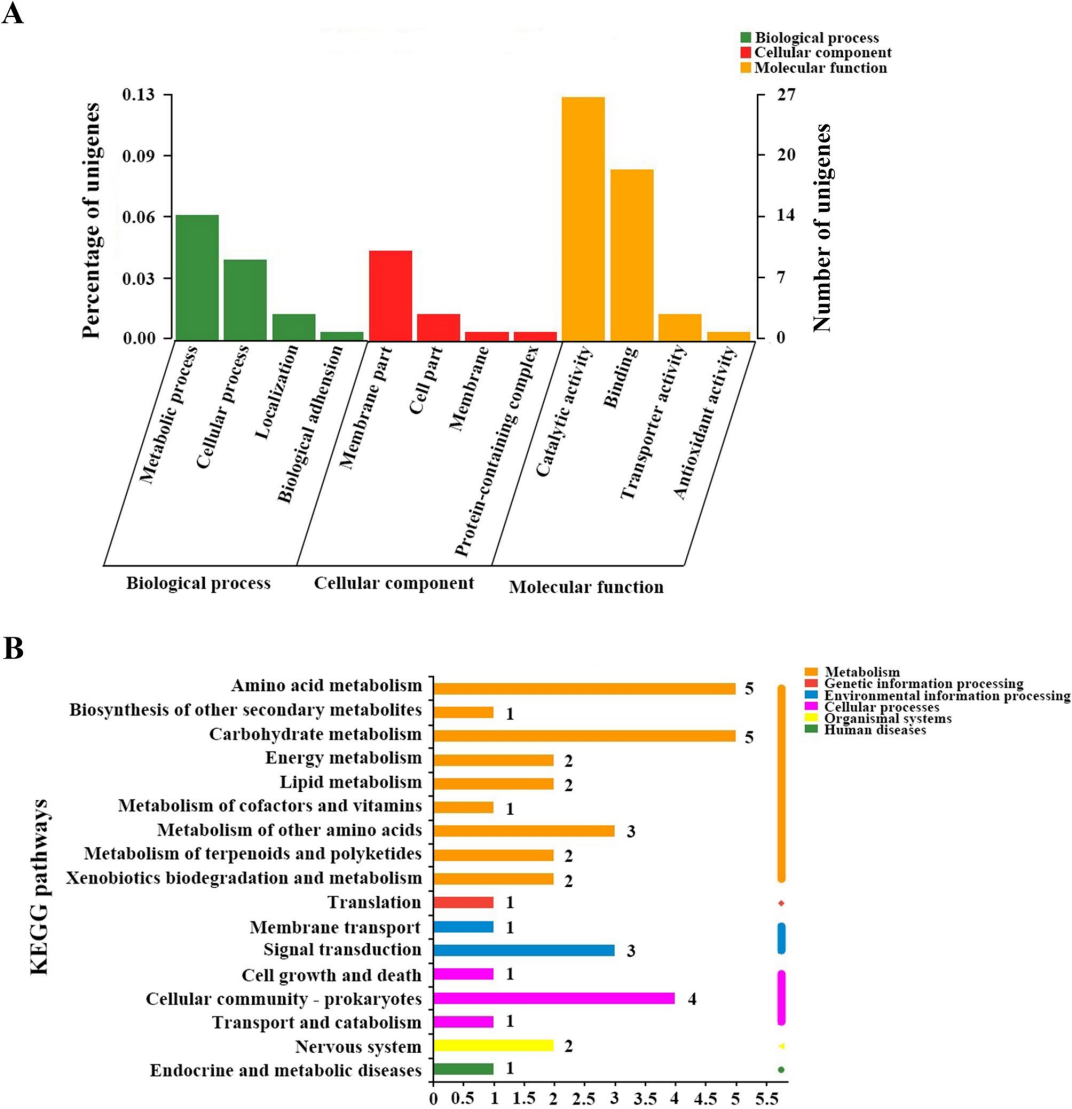
Analysis of the genes only expressed in spinosad high-yield strain *S. spinosa* S3-3 using GO annotation and KEGG annotation. (**A**) Analysis of the genes only expressed in spinosad high-yield strain *S. spinosa* S3-3 using GO annotation; (**B**) Analysis of the genes only expressed in spinosad high yield strain *S. spinosa* S3-3 using KEGG annotation. (The chart was carried out using Majorbio Cloud Platform. URL: http://www.majorbio.com).

To figure out the trend and characteristic of genes expressed in *S. spinosa* S3-3, the transcriptomic libraries of *S. spinosa* ATCC_49460 and *S. spinosa* S3-3 at different time point were compared and the genes uniquely expressed in *S. spinosa* S3-3 were annotated using GO and KEGG databases. Taking all the data into account, 5613 genes were expressed in both strains throughout the fermentation period (Figure S2A). Then at each time point, the intersection of the genes expressed in both strains was established and 6088, 5955 and 6309 genes were included in the third day, the fifth day and the seventh day, respectively (Figure S2B). Additionally, there were still lots of genes that were not included in the intersection mentioned above. Here, we took the genes only expressed in *S. spinosa* S3-3 as the candidate genes to have a relationship with spinosad synthesis. By summarizing the genes only expressed in *S. spinosa* S3-3 at all three time points, 152 genes were selected (Figure S2C). The largest proportion of these genes were annotated as four categories according to GO rules: catalytic activity (molecular function), binding (molecular function), metabolic process (biological process) and membrane part (cell component) category according to GO rules (Fig. 2A). As for KEGG annotation, 17 of these genes were annotated and classified, most of them were suggested to take part in amino acid metabolism (including amino acid metabolism and other amino acid metabolism), carbohydrate metabolism (including carbohydrate metabolism, lipid metabolism, terpenoids and polyketides metabolism) and cellular community pathway (Fig. 2B).

### Analysis of differentially expressed genes (DEGs) between *S. spinosa* ATCC_49460 and *S. spinosa* S3-3 during fermentation process

**Figure 3:**
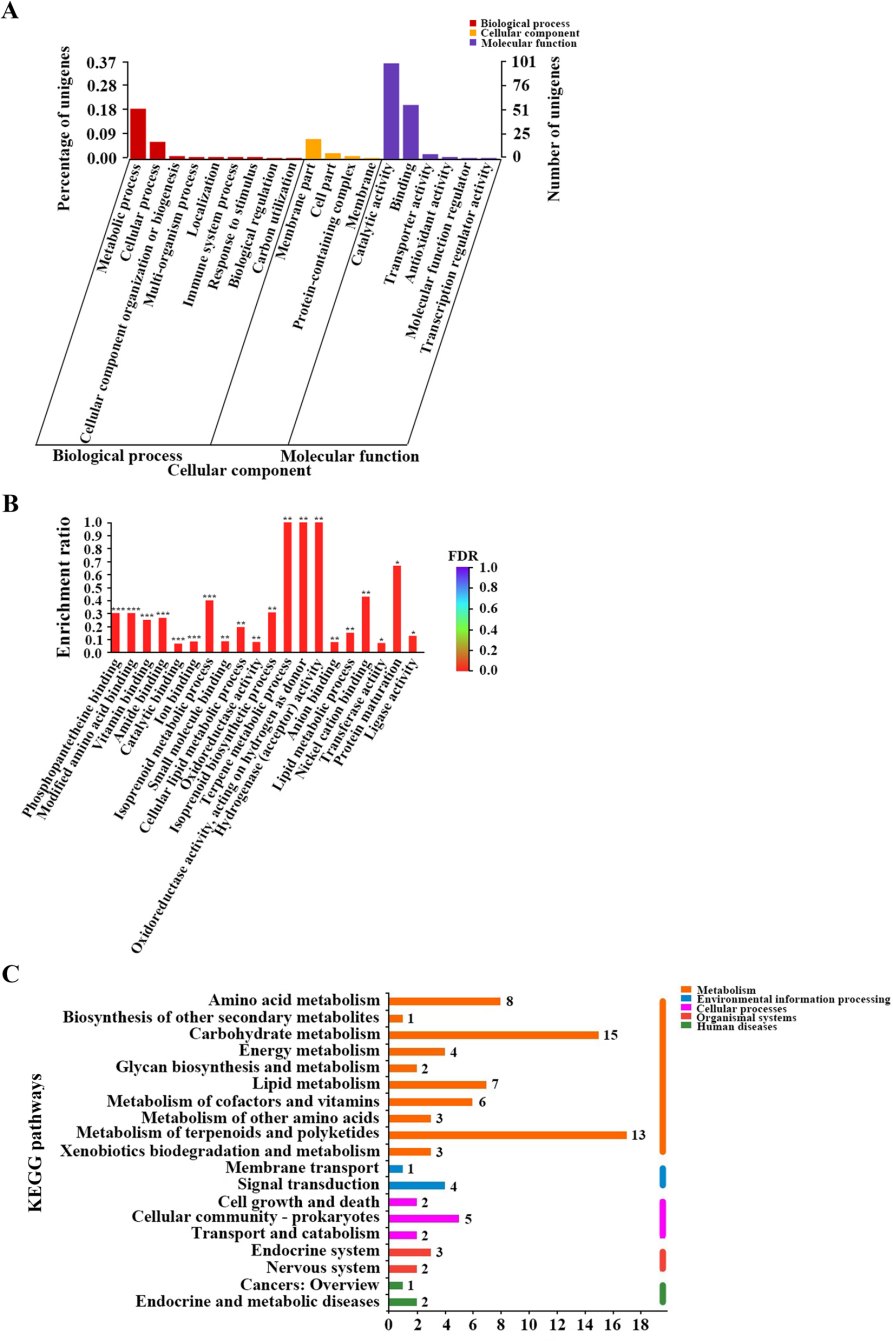
Annotation and enrichment of the up-regulated genes in *S. spinosa* S3-3. (**A**) GO annotation of the up-regulated genes in *S. spinosa* S3-3; (**B**) GO enrichment of the up-regulated genes in *S. spinosa* S3-3; (**C**) Annotation of the up-regulated genes in *S. spinosa* S3-3 using KEGG database. (The chart was carried out using Majorbio Cloud Platform. URL: http://www.majorbio.com).

To analyze the differentially expressed genes between *S. spinosa* ATCC_49460 and *S. spinosa* S3-3, the transcriptional libraries of the two strains were compared at each time point, and three sets of differentially expressed genes with an adjusted *p*-value < 0.05 and fold-change > 5 or < 0.2 were summarized. Comparing with *S. spinosa* ATCC_49460, 942, 963 and 430 genes were up-regulated, while 350, 188 and 285 genes were down-regulated, respectively in the *S. spinosa* S3-3 strain. Furthermore, 275 and 70 genes were confirmed to be up or down regulated during all the three fermentation periods, respectively (Figure S3). Then the up-regulated genes were annotated and classified according to GO classification system, showing that 101 genes belonged to catalytic activity category (molecular function), 57 genes belonged to binding category (molecular function), 53 genes belonged to metabolic process category (biological process) and 21 genes belonged to membrane part category (cell component) (Fig. 3A). By GO enrichment, the most affected GO terms were phosphopantetheine binding, modified amino acid binding, vitamin binding, amide binding, ion binding and isoprenoid metabolic process (FDR < 0.001) (Fig. 3B). Following by the adaption of the up-regulated genes to KEGG pathways, it was suggested that the drastically changed pathways were the metabolism of terpenoids and polyketides, carbohydrate metabolism, amino acid metabolism and lipid metabolism pathways (Fig. 3C).

### Measurement of the expression levels of genes located in spinosyns biosynthesis gene clusters

**Figure 4:**
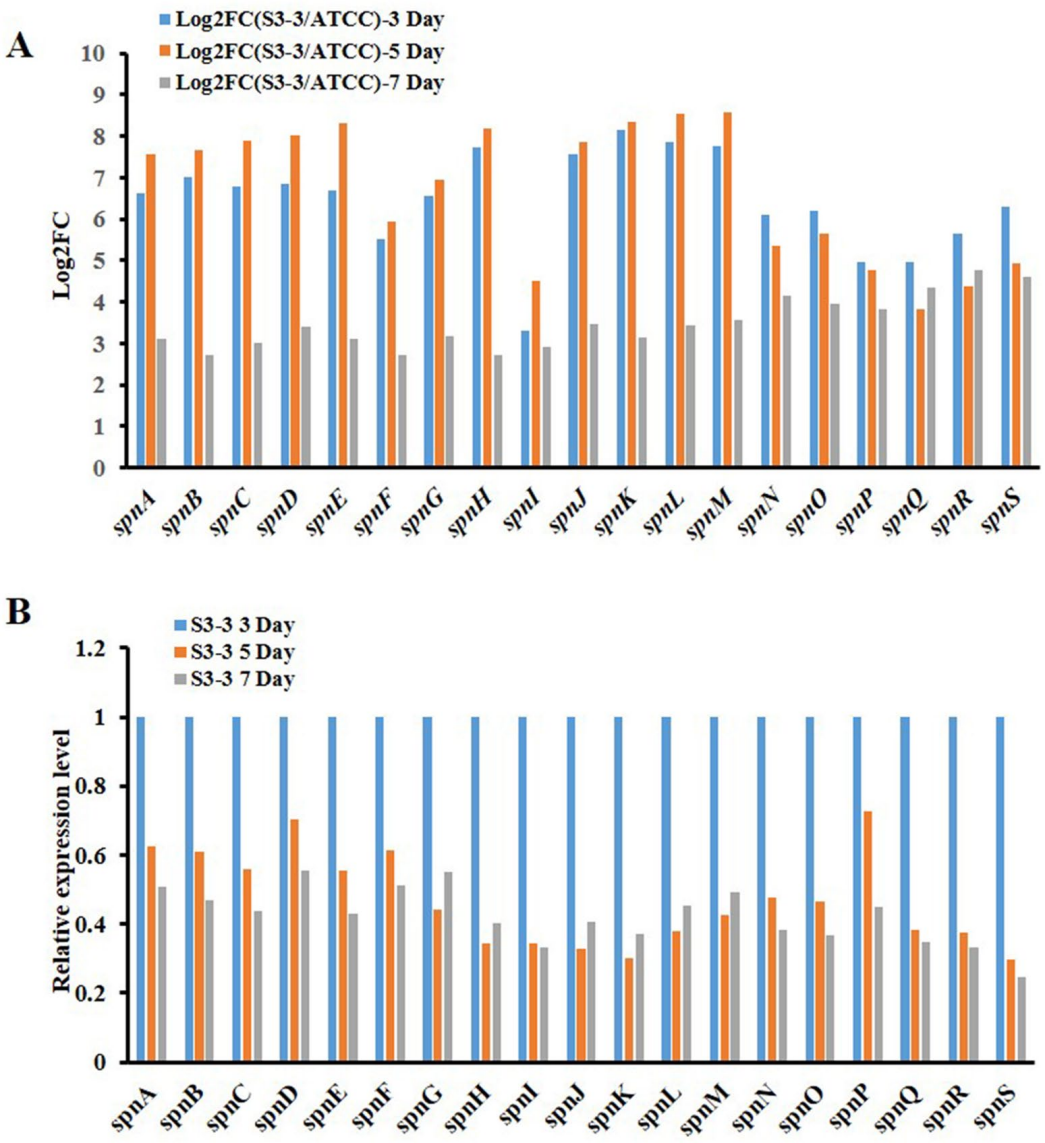
Transcriptional analysis of spinosyns synthesis genes in *S. spinosa* S3-3. (**A**) The expression differences of genes located in spinosyns biosynthetic gene cluster between *S. spinosa* ATCC_49460 and *S. spinosa* S3-3. The expression level of genes in *S. spinosa* ATCC_49460 was set as 1. (**B**) The expression differences of genes located in spinosyns biosynthetic gene cluster between different time points in *S. spinosa* S3-3. The expression level of genes at 3 day was set as 1. (The chart was carried out using Microsoft Office Professional Plus 2013).

The spinosyns biosynthesis gene cluster, which spanned 74 kilo-base and contained 19 genes, played an essential role in the synthesis of spinosad^8^. The function of these genes mainly included polyketide synthesis, intramolecular cycloaddition, deoxysugars addition and so on. At this section, the expression levels of all 19 genes were compared between *S. spinosa* ATCC 49460 and *S. spinosa* S3-3. It was suggested that all genes involved in the spinosad synthesis were up-regulated at least 6.591 folds in *S. spinosa* S3-3 compared with *S. spinosa* ATCC_49460 at all three fermentation time points. At late lag phase and middle logarithmic phase, *spnA*, *B*, *C*, *D*, *E*, *H*, *J*, *K*, *L* and *M* were up-regulated at highest level (log2FC > 7), which were mostly related to polyketide synthesis, intramolecular carbon–carbon bond formation. Though two of the genes related to rhamnose modification, *spnH* and *spnK*, were greatly up-regulated, the first gene involved in this process, *spnI*, was not up-regulated high enough to supply the substrate needed. Additionally, *spnO, N, P*, *Q, R* and S were also suggested to be at a relatively low transcriptional level, indicating that the forosamine biosynthetic pathway and glycotransferase activity could also be a lagging process during spinosad synthesis (Fig. 4A, Table 2). Interestingly, the transcriptional level of almost all the *spn* genes drop to 50% at logarithmic phase and stationary phase comparing to that at the late lag phase (Fig. 4B, Table 3), indicating the construction of spinosad biosynthesis system is mainly at the late lag phase or early logarithmic phase.

**Table 2:** The expression levels of genes located in spinosad biosynthetic gene cluster in *S. spinosa* S3-3 compared to *S. spinosa* ATCC_49460 at different time points.

Gene name	log2FC (3 day)	log2FC (5 day)	log2FC (7 day)	Gene name	log2FC (3 day)	log2FC (5 day)	log2FC (7 day)	Gene name	log2FC (3 day)	log2FC (5 day)	log2FC (7 day)
*spnA*	6.63	7.58	3.11	*spnH*	7.73	8.17	2.72	*spnO*	6.20	5.65	3.97
*spnB*	7.01	7.67	2.70	*spnI*	3.32	4.51	2.92	*spnP*	4.97	4.77	3.84
*spnC*	6.78	7.88	3.02	*spnJ*	7.58	7.87	3.48	*spnQ*	4.98	3.82	4.33
*spnD*	6.84	8.02	3.39	*spnK*	8.15	8.35	3.15	*spnR*	5.64	4.38	4.76
*spnE*	6.67	8.31	3.11	*spnL*	7.86	8.55	3.43	*spnS*	6.30	4.93	4.61
*spnF*	5.52	5.94	2.73	*spnM*	7.75	8.57	3.58				
*spnG*	6.57	6.96	3.18	*spnN*	6.09	5.35	4.14

**Table 3:** The expression differences of genes located in spinosad biosynthetic gene cluster between different time points in *S. spinosa* S3-3.

Gene name	log2FC (5 day/3 day)	log2FC (7 day/3 day)	Gene name	log2FC (5 day/3 day)	log2FC (7 day/3 day)	Gene name	log2FC (5 day/3 day)	log2FC (7 day/3 day)
*spnA*	− 0.68	− 0.98	*spnH*	− 1.53	− 1.31	*spnO*	− 1.10	− 1.45
*spnB*	− 0.71	− 1.08	*spnI*	− 1.54	− 1.59	*spnP*	− 0.46	− 1.15
*spnC*	− 0.84	− 1.20	*spnJ*	− 1.60	− 1.30	*spnQ*	− 1.38	− 1.53
*spnD*	− 0.51	− 0.85	*spnK*	− 1.74	− 1.43	*spnR*	− 1.41	− 1.59
*spnE*	− 0.85	− 1.22	*spnL*	− 1.40	− 1.15	*spnS*	− 1.75	− 2.02
*spnF*	− 0.70	− 0.96	*spnM*	− 1.23	− 1.02			
*spnG*	− 1.18	− 0.86	*spnN*	− 1.07	− 1.38			

### Analysis of the transcriptional levels of genes related to primary metabolic pathways

The biosynthesis of spinosad showed a close relationship with the cell primary metabolic pathways. Hence, the transcriptomic data concerning glycometabolism, fatty acid metabolism and amino acid metabolism were discussed in this section.

#### Glycometabolic pathway

**Figure 5:**
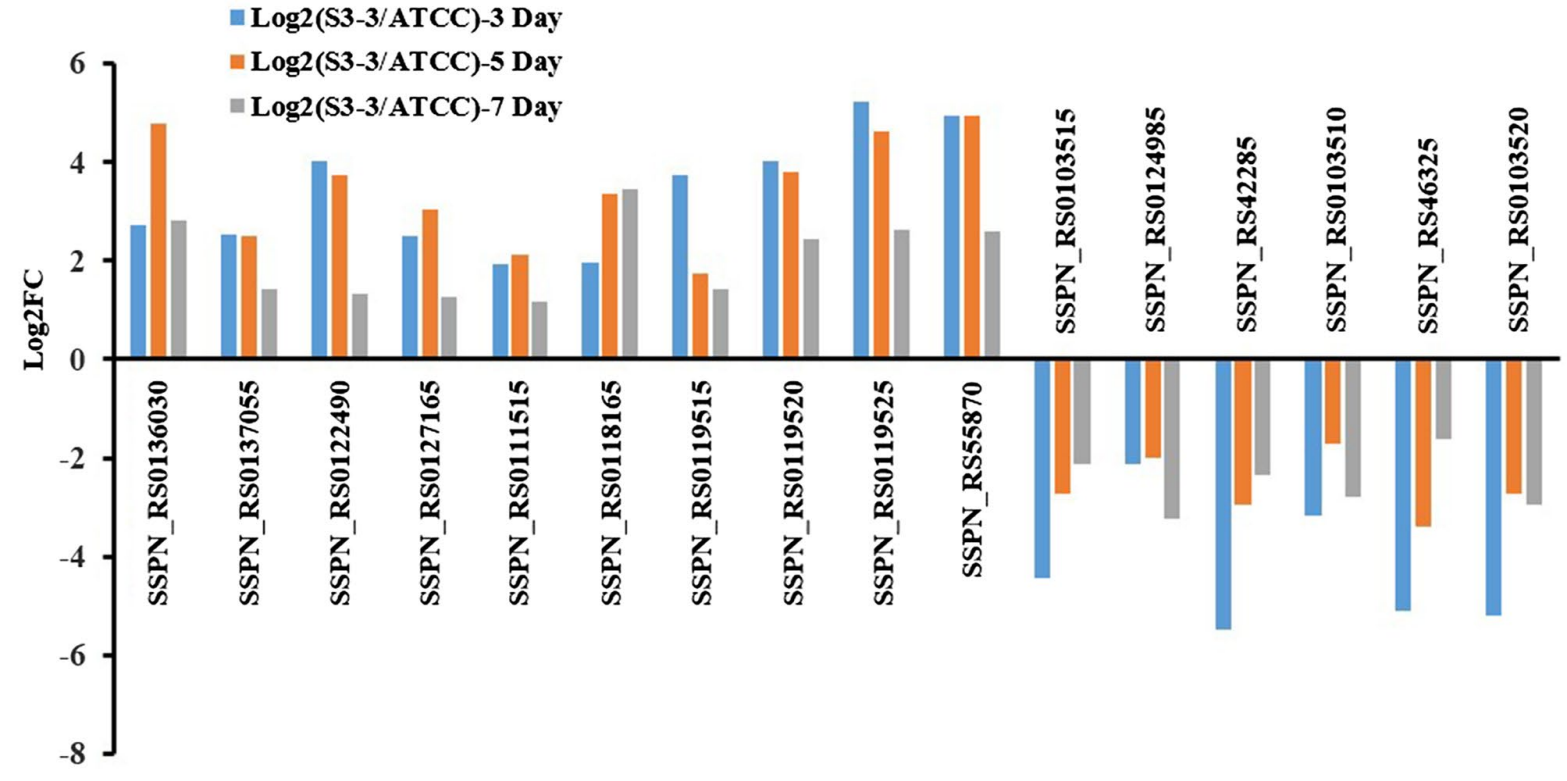
Transcriptional analysis of the genes related to glycometabolism pathway. The expression level of genes in *S. spinosa* ATCC_49460 was set as 1. (The chart was carried out using Microsoft Office Professional Plus 2013).

In bacteria, glycometabolic pathway is the most important metabolic pathway, for it supplies the energy and reduce power cell needed. In this part, the genes involved in glycolysis process, gluconeogenesis process, pyruvate metabolism process and citrate cycle were taken into consideration to distinguish the metabolic specialty in *S. spinosa* S3-3. By comparing the transcriptomic data of the two strains at three fermentation periods, 10 genes, including a glucose-6-phosphate isomerase (locus SSPN_RS0136030), two aldehyde dehydrogenases (locus SSPN_RS0137055 and SSPN_RS0122490), a phosphoglycerate kinase (locus SSPN_RS0127165), a phosphoenolpyruvate carboxykinase (locus SSPN_RS0111515) and five dehydrogenases (locus SSPN_RS0118165, SSPN_RS0119515, SSPN_RS0119520, SSPN_RS0119525 and SSPN_RS55870), were up-regulated at least 2.2 folds (log2FC > 1) in *S. spinosa* S3-3 than in *S. spinosa* ATCC_49460. However, the metabolism of pyruvate was seriously impaired, as five genes involved in pyruvate dihydrogen process (locus SSPN_RS0103515, SSPN_RS0124985, SSPN_RS42285, SSPN_RS0103510 and SSPN_RS46325) were depressed (log2FC < -1). Considering that the oxaloacetate molecules were transformed to phosphoenolpyruvate (PEP) by phosphoenolpyruvate carboxykinase through gluconeogenesis process, all these results indicated a metabolic flux towards the generation of 3-phosphoglycerate (3-PG), pyruvate and reducing power. At last, the transcription of α-ketoacid dehydrogenase β subunit (locus SSPN_RS0103520) was also depressed dramatically, indicating α-ketoglutarate may be accumulated and transformed to glutamic acid in the impaired pathway (Fig. 5).

#### Fatty acid metabolic pathway

**Figure 6:**
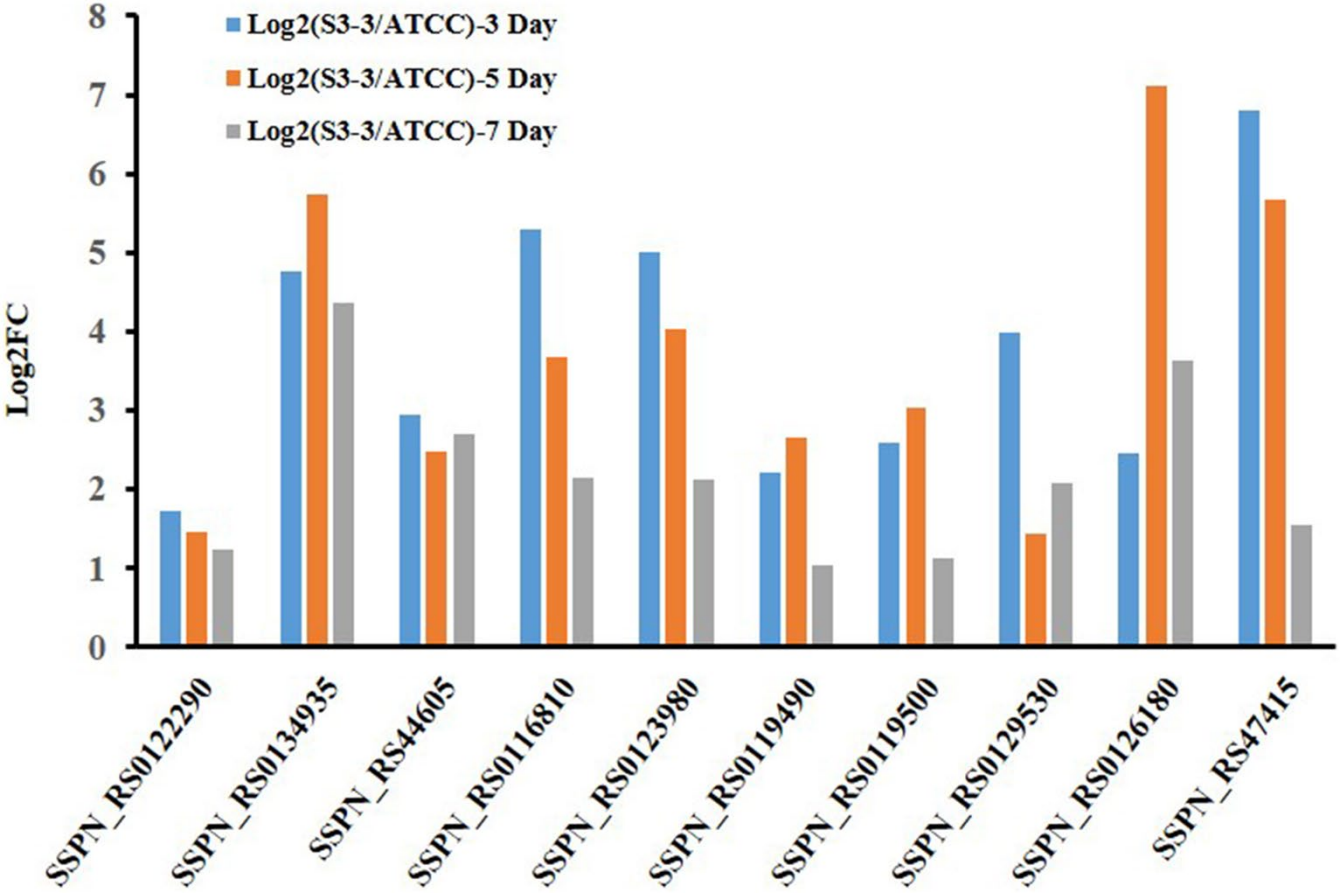
Transcriptional analysis of the genes related to fatty acid metabolism pathway. The expression level of genes in* S. spinosa* ATCC_49460 was set as 1. (The chart was carried out using Microsoft Office Professional Plus 2013).

In *S. spinosa* strains, spinosad polyketide chain formation begins from propionic acid and extended with the addition of acetyl and propionyl residues. During fermentation process, soybean oil was added into the fermentation medium to enhance the production of spinosad, indicating that fatty acid metabolism played a key role in the spinosad biosynthesis process^13^. According to the fatty acid metabolic pathway in *S. spinosa* S3-3, 10 key genes were expressed at least onefold higher than that in *S. spinosa* ATCC_49460. Acyl-CoA dehydrogenase (locus SSPN_RS0122290) took part in the process of fatty acid β-oxidation, supplying acetyl-CoA, which was transformed to malonyl-CoA by an acetyl-CoA carboxylase (Locus SSPN_RS0134935). Two β-ketoacyl synthase (locus SSPN_RS44605 and SSPN_RS0116810) controlling the biosynthesis of ketoacyl, was the basic enzyme in fatty acid or polyketide formation process. An acyl carrier protein (locus SSPN_RS0123980) synthesized the transport protein. Three ketoacyl-ACP synthase III (locus SSPN_RS0119490, SSPN_RS0119500 and SSPN_RS0129530) loaded ketoacyl to acyl carrier protein, preparing for the condensation process. An AMP-binding protein (locus SSPN_RS0126180) and an adenylyltransferase (locus SSPN_RS47415) helped to reduce-power delivery. In conclusion, the fatty acid β-oxidation process and polyketide synthesis process were extremely enhanced in *S. spinosa* S3-3 (Fig. 6).

#### Amino acid metabolism

**Figure 7:**
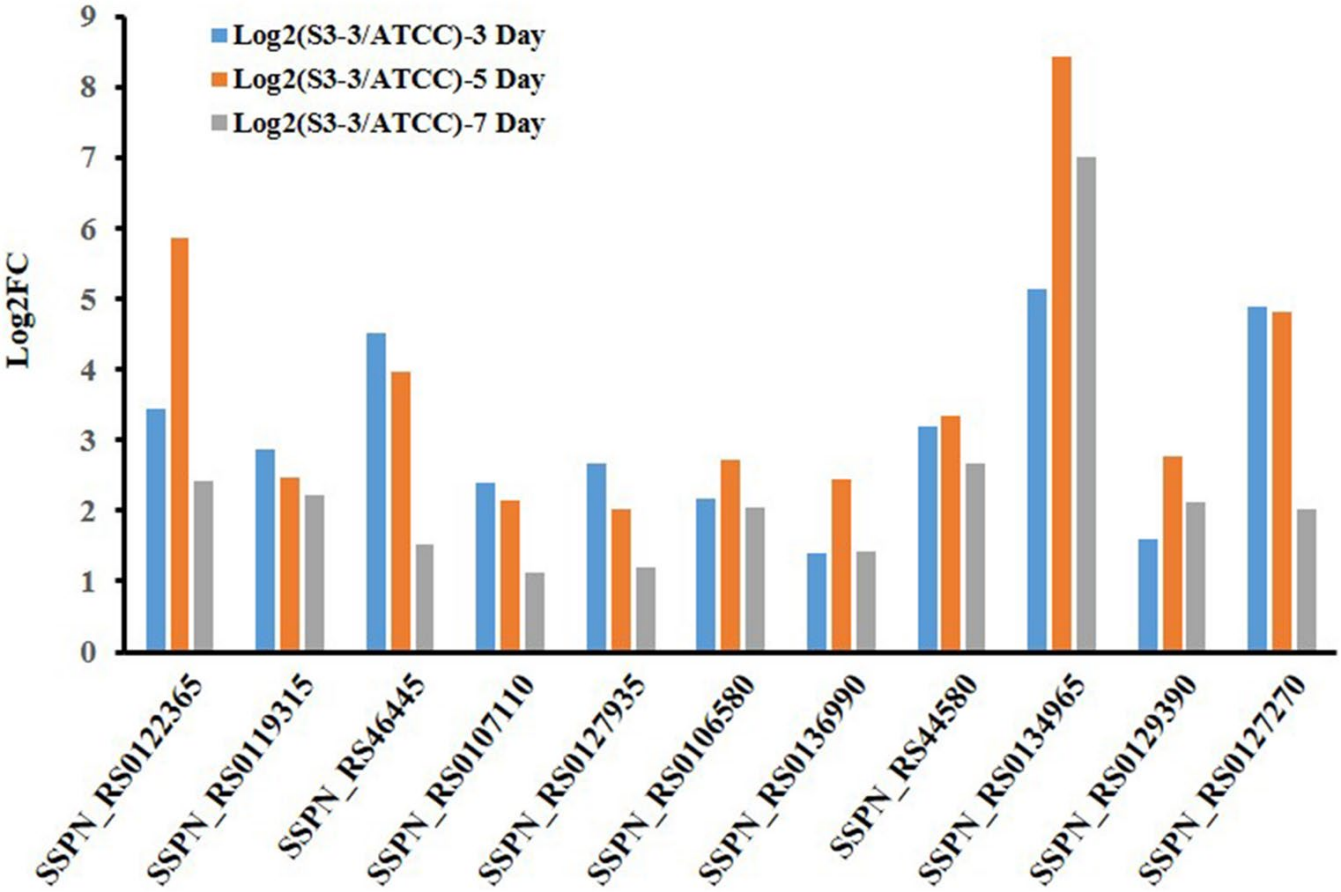
Transcriptional analysis of the genes related to amino acids metabolism pathway. The expression level of genes in *S. spinosa *ATCC_49460 was set as 1. (The chart was carried out using Microsoft Office Professional Plus 2013).

Since amino acids could directly join the antibiotics biosynthesis process as important biological small molecules^29,37^, we traced the transcriptional levels of genes related to amino acid metabolism in the both *S. spinosa* strains. In *S. spinosa* S3-3 aromatic amino acid lysis metabolism was enhanced, as the transcriptional levels of two aromatic amino acid lyase (locus SSPN_RS0122365 and SSPN_RS0119315), a tryptophanase (locus SSPN_RS46445) and a tryptophan-dioxygenase (locus SSPN_RS0107110) were up-regulated. And the aliphatic acid metabolism, especially glutamate metabolism and aspartate metabolism, should be the core pathway engaged in spinosad synthesis. In *S. spinosa* S3-3, three aspartate metabolism related genes (locus SSPN_RS0127935, SSPN_RS0106580 and SSPN_RS0136990) and three glutamate metabolism related genes (locus SSPN_RS44580, SSPN_RS0134965 and SSPN_RS0129390) were up-regulated. Additionally, the isocitrate dehydrogenase (locus SSPN_RS0127270) was up regulated to supply the substrate for aliphatic acid synthesis (Fig. 7).

## Discussion

Spinosad, an environmentally friendly insecticide, has a desired business prospect, which attracted great research attention to track its biosynthesis process and to improving its production yield. Since 1990s, lots of work has been applied, including mutagenesis, gene shuffling, spinosad heterologous expression, fermentation optimization, as well as proteomics analyzation^26,38–42^. However, few transcriptional level attempts have been done to reveal the differentially expressed genes between wild-type strain and spinosad high-yield strain during fermentation periods^35^. In this study, the wild-type strain (*S. spinosa* ATCC_49460) and spinosad high-yield strain (*S. spinosa* S3-3) were sampled at late lag phase, middle logarithmic phase and early stationary phase to explore characteristics of the gene transcription. The RNA-Seq based analysis revealed the up-regulated pathway in *S. spinosa* S3-3 strain and shed new light on *S. spinosa* genetic engineering.

Depending on high-throughput sequencing techniques, the mRNA and rRNA of 12 samples, including two strains at three time points with two biological repeats, were analyzed. At Q20 level, at least 98.12% of the sequenced reads were identified as clean reads, indicating the high quality of the libraries. Though 7301 genes were annotated in the whole genome, 2660 genes were classified by GO annotation and 2659 genes were classified according to KEGG annotation, which suggested the poor understanding of the hereditary information of *S. spinosa*. Therefore, the analyzation of the transcriptome data was limited, and re-sequencing and re-annotation of the *S. spinosa* genome should be the most emergency task in the next step. By comparing the transcriptome libraries between the two strains through the three fermentation periods, 152 genes were found to only express in *S. spinosa* S3-3 and 275 genes were found to be up-regulated in *S. spinosa* S3-3. By GO classification, most of these differentially expressed genes were in catalytic activity, metabolic process and membrane part term, suggesting these functions have the maximum association with spinosad biosynthetic pathway.

Spinosad biosynthetic gene cluster was proved to be the most important gene cluster. And the heterologous expression of both spinosad biosynthesis cluster and rhamnose synthesis genes in *Streptomyces coelicolor* resulted in the production of 1 or 1.5 μg/ml spinosad intracellularly^28^. Here, the transcriptional data of this significant genes were analyzed to identify the speed limit procedure during spinosad production process. It showed that the genes involved in carbon skeleton formation, including PKS formation and intramolecular carbon–carbon bond formation, were up-regulated at a maximum stage (change fold > 100). Comparing to these genes, the least up-regulated gene was *spnI*, which involved in rhamnose O-methylation. Furthermore, genes related to forosamine synthesis and glycol-transfer process, *spnN, O, P, Q, R* and *S* also showed a relatively low expression level. According to the previous research, the intermediate, spinosyn P-CH2, was found to accumulate, which indicated that the enzymatic activity of SpnI was insufficient in the spinosad heterologous expression strains^44^. Additionally, the over-expression of *spnI* and *spnN* resulted in the great enhancement of spinosad production^44^. In this study, the result reflected the up regulation level of *spnI, N, O, P, Q, R* and *S* were not high enough comparing to the other spinosad synthesis related gene, indicating these gens could still be the bottle neck in the spinosad production process, which was consistent with the previous reports.

The spinosad production process was suggested to start from a propionic acid residue followed by elongation of the carbon skeleton with addition of acetyl residue and propionyl residue, which indicated the strong relationship between carbon metabolism and spinosad formation. During fermentation assays, starch was added as delayed carbon resource to supply the substrates besides glucose. According to previous research, phosphoenolpyruvic phosphonomutase was mutated to block the replenishment pathway, resulting in the enhancement of the accumulation of pyruvate and the production of spinosad^35^. Moreover, the addition of soybean oil in the culturing medium elevated the β-oxidation enzyme activity in fatty acid degradation pathway, which supplied more acetyl-CoA and leveled up spinosad production^13^. By genome-scale metabolic network reconstruction, a few amino acids were considered to directly participate in the biosynthesis of antibiotics as the precursor^43^. In this work, the expression levels of genes involved in glycometabolic pathway, fatty acid metabolic pathway and amino acid metabolic pathway were examined to reveal the metabolic relationship between primary metabolism and spinosad production. Among glycometabolic pathway, the glycolysis pathway (including glucose-6-phosphate isomerase, aldehyde dehydrogenase and phosphoglycerate kinase) was activated greatly, the expression of four pyruvate dehydrogenase were inhibited, implying the accumulation of phosphoenolpyruvic and pyruvate. To fulfill the shortage of acetyl-CoA, the fatty acid β-oxidation pathway was activated. Also, the up-regulation of acyl carrier protein, β-ketoacyl synthase and ketoacyl-ACP synthase showed a high level of acyl supplement intracellularly, which might participate in the spinosad biosynthesis process. Different from the genome-scale metabolic network reconstruction results, the two acidic amino acid, glutamic acid and aspartic acid, was at the central of differentially expressed pathways, reflecting the addition of these two amino acids might have a help in spinosad production.

## Conclusion

**Figure 8:**
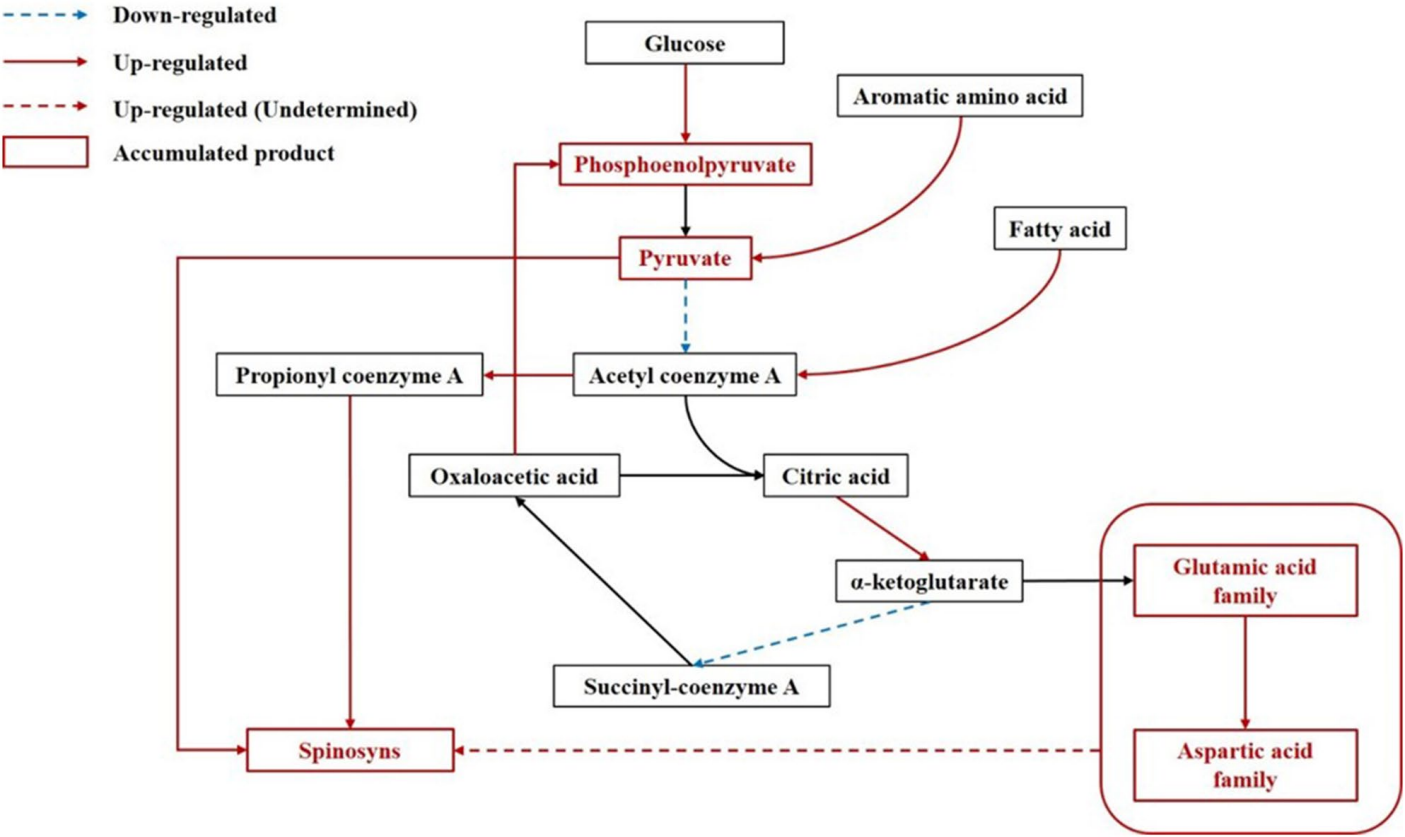
Schematic metabolism network between spinosad biosynthesis and primary metabolism pathways. (The chart was carried out using Microsoft Office Professional Plus 2013).

The present study analyzed the genes differentially expressed in wild-type strain *S. spinosa* ATCC_49460 and spinosad high-yield strain *S. spinosa* S3-3. By comparing the transcriptomic libraries, the genes of *spnI*, *N, O, P, Q, R* and *S* were considered as the bottleneck during spinosad formation. Additionally, the cell primary metabolism was supposed to have a close relationship with spinosad biosynthesis. In carbon metabolic pathways, the glycolysis was enhanced by the up regulation of glucose-6-phosphate isomerase, aldehyde dehydrogenase and phosphoglycerate kinase. Pyruvate and PEP were accumulated with the decreased expression of pyruvate dehydrogenases and the activation of phosphoenolpyruvate carboxykinase (GTP). According to fatty acid metabolism pathway, the fatty acid β-oxidation pathway, ketoacyl synthesis pathway and acyl transport pathway were up-regulated, that supplied enough amount of acyl residues for polyketide synthesis. Furthermore, glutamic acid and aspartic acid were proved to be at the central of amino acid in metabolism networks, constructing another way to supply the carbon residues. Taken together, several metabolic pathways were alerted to adapt for the great enhancement of spinosad production (Fig. 8). In conclusion, this work firstly compared the transcriptomic libraries between *S. spinosa* wild-type strain and spinosad high-yield strain and revealed the relationship between primary metabolic pathway and spinosad biosynthesis pathway, suggesting directions of molecular engineering for enhancement of spinosad production.

## Materials and methods

### Strains and cultivation

The wild-type strain, *S. spinosa* ATCC_49460 was purchased from China General Microbiological Culture Collection Center (CGMCC). The high-yield mutant, *S. spinosa* S3-3 derived from strain *S. spinosa* ATCC_49460, was obtained by several rounds of chemical mutagenesis.

For strain activation, 0.5 mL of spore suspensions (approx. 10^7^–10^8^ spores/mL) was inoculated to seed medium, and incubated for 54 h at 29 °C, 240 rpm on a rotary shaker. Each liter of seed medium contained: 4 g yeast extract, 4 g tryptone, 4 g casein hydrolysate (acid), 10 g glucose, 1.36 g K_2_HPO_4_, 0.5 g MgSO_4_, with a pH of 7.2 ± 0.1 before autoclaving. Next, 10% (v/v) of the seed culture was inoculated into 30 mL of seed medium to prepare fermentation seed liquid. Then, 3 mL of seed culture (10%, v/v) was inoculated into 30 mL of fermentation medium in 250-mL Erlenmeyer flask and cultured for 7 d at 29 °C, 240 rpm. The composition of the fermentation medium (per liter) was: 10 g peptonized milk, 20 g cottonseed protein, 20 g dextrin, 1 g NaCl, 1 g MgSO_4_, 0.5 g KH_2_PO_4_, 3 g CaCO_3_, 10 g yeast extract, 60 g glucose, 20 g soybean oil. The pH value was adjusted to 7.2 with 1 M of NaOH before autoclaving. All experiments had three biological replicates.

### Analysis of spinosad production

The yield of spinosad was assessed using HPLC as described by Huang et al.^13^. For determination of spinosad productivity, fermentation broth was extracted with 3 × volume of methanol overnight at 4 °C without exposing to light. Then, the leaching liquor was centrifuged at 14,000 rpm for 15 min before being analyzed by HPLC. Samples (10 μL) were injected into an Agilent C18 reverse-phase column (5 μm, 150 × 4.6 mm) operated at 40 °C using an isocratic mobile phase composed of 45% (v/v) methanol, 45% (v/v) acetonitrile, and 10% 6.5 mM ammonium acetate with a flow rate of 1.0 mL/min using a UV detector at 244 nm.

### RNA extraction

Total RNA was extracted from the samples using TRIzol Reagent according to the manufacturer’s instructions (Invitrogen) and genomic DNA was removed using *DNase* I (Takara). Then RNA quality was determined by 2100 Bioanalyser (Agilent) and quantified using the ND-2000 (NanoDrop Technologies). Only high-quality RNA sample (OD260/280 = 1.8–2.0, OD260/230 ≥ 2.0, RIN ≥ 6.5, 28S:18S ≥ 1.0, ≥ 100 ng/μl, ≥ 2 μg) was used to construct sequencing library.

### Library construction and sequencing

RNA-seq transcriptome library was prepared with TruSeqTM RNA sample preparation Kit from Illumina (San Diego, CA) using 2 μg of total RNA. Shortly, ribosomal RNA (rRNA) depletion instead of poly(A) purification is performed by Ribo-Zero Magnetic kit (epicenter) and then all mRNAs were broken into short (200 nt) fragments by adding fragmentation buffer firstly. Secondly double-stranded cDNA was synthesized using a SuperScript double-stranded cDNA synthesis kit (Invitrogen, CA) with random hexamer primers (Illumina). When the second strand cDNA was synthesized, dUTP was incorporated in place of dTTP. Then the synthesized cDNA was subjected to end-repair, phosphorylation and ‘A’ base addition according to Illumina’s library construction protocol. The second strand cDNA with dUTP was recognized and degraded by UNG enzyme. Libraries were size selected for cDNA target fragments of 200 bp on 2% Low Range Ultra Agarose followed by PCR amplified using Phusion DNA polymerase (NEB) for 15 PCR cycles. After quantified by TBS380, paired-end RNA-seq sequencing library was sequenced with the Illumina HiSeq×TEN (2 × 150 bp read length).The processing of original images to sequences, base-calling, and quality value calculations were performed using the Illumina GA Pipeline (version 1.6), in which 150 bp paired-end reads were obtained. A Perl program was written to select clean reads by removing low-quality sequences, reads with more than 5% of N bases (unknown bases) and reads containing adaptor sequences.

### Data analysis

The overall comparation of transcriptomic data between different *S. spinosa* strains were carried out using the free online platform of Majorbio Cloud Platform (http://www.majorbio.com). The KEGG annotation and enrichment were undertaken depending on the KEGG website (https://www.kegg.jp/kegg/)^45–47^ The histogram and schematic graph concerning the relationships between primary metabolism pathway and spinosad production were constructed using Microsoft Office Excel and Powerpoint (Version Professional Plus 2013).

## Supplementary Information

Supplementary Figures.
