# Supplementary material for: Comparative transcriptomic analysis of two Saccharopolyspora spinosa strains reveals the relationships between primary metabolism and spinosad production
Source: Sci Rep. 2021 Jul 20;11:14779. doi: 10.1038/s41598-021-94251-z (PMC8292330; doi:10.1038/s41598-021-94251-z)
Supplement: Supplementary file 1 — Supplementary Figures. [file 41598_2021_94251_MOESM1_ESM.docx]

**Supplementary Information**





Figure S1. Analysis of the spinosad production in S. spinosa ATCC_49460 and S3-3.


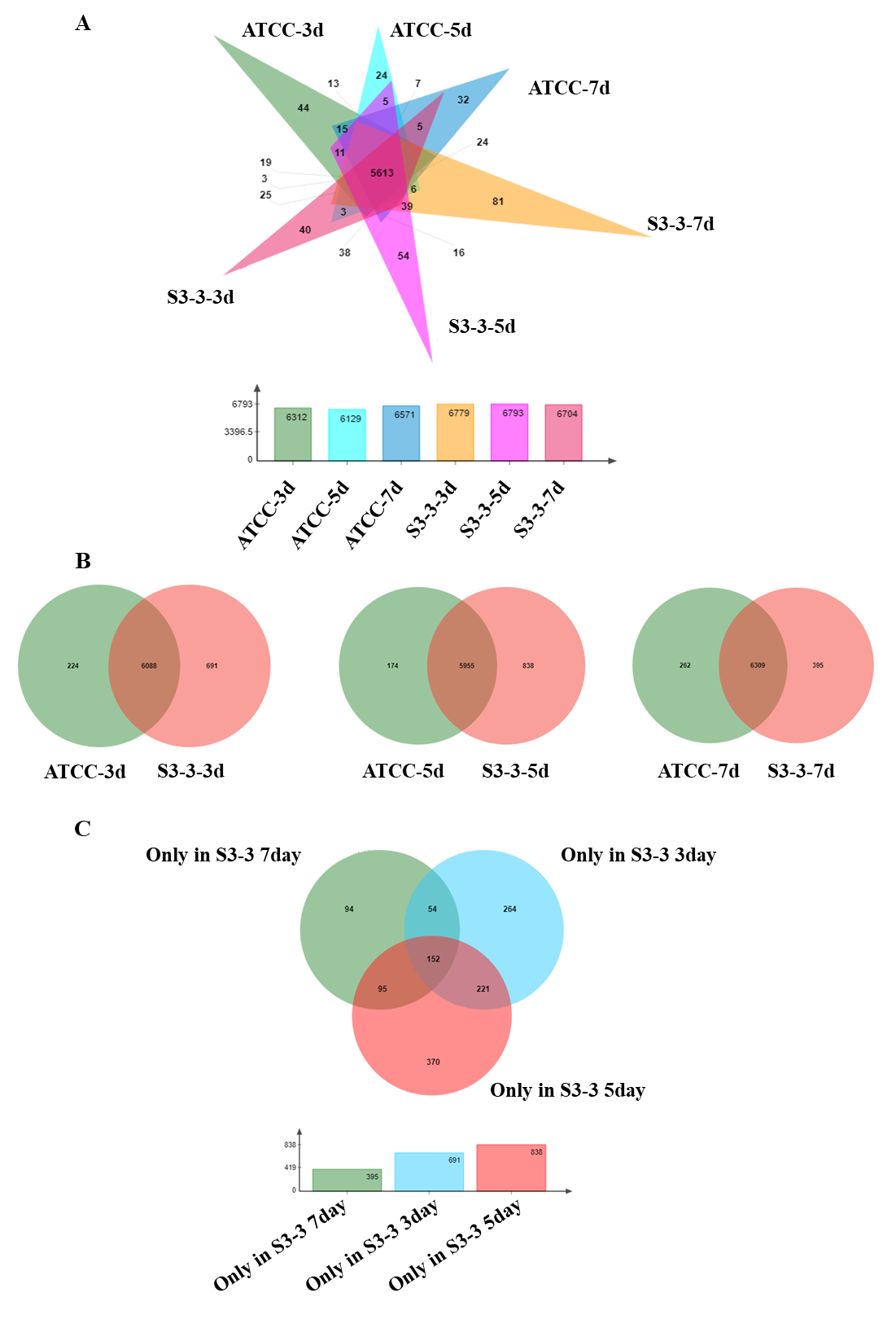
Figure S2. The identification of the genes specifically expressed in S. spinosa S3-3 during fermentation periods.


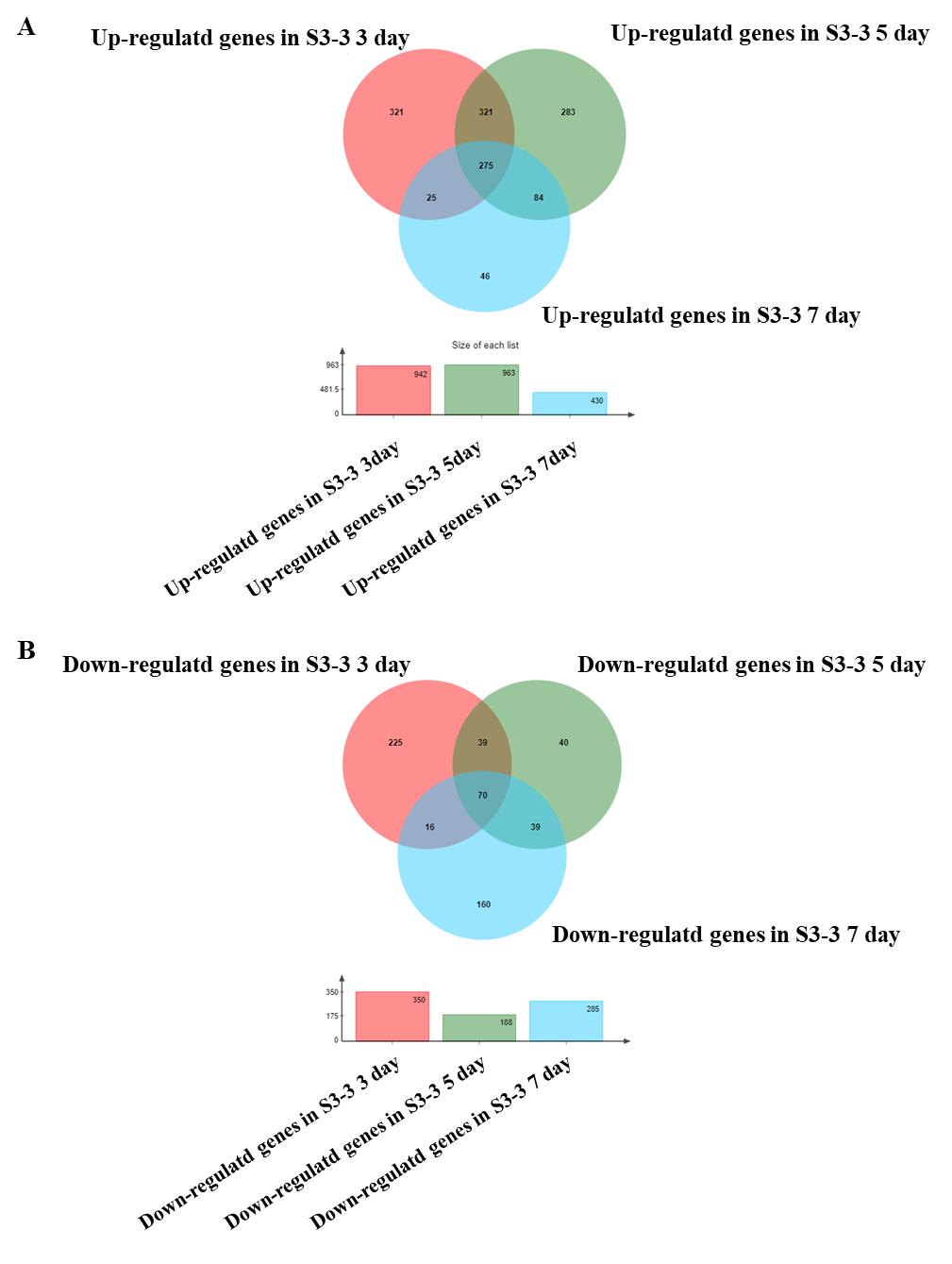


Figure S3. The identification of genes differentially expressed in S. spinosa S3-3.
